# Aldosterone-Regulating Receptors and Aldosterone-Driver Somatic Mutations

**DOI:** 10.3389/fendo.2021.644382

**Published:** 2021-03-16

**Authors:** Jung Soo Lim, Samuel W. Plaska, Juilee Rege, William E. Rainey, Adina F. Turcu

**Affiliations:** ^1^ Department of Molecular and Integrative Physiology, University of Michigan, Ann Arbor, MI, United States; ^2^ Division of Endocrinology and Metabolism, Department of Internal Medicine, Yonsei University Wonju College of Medicine, Wonju Severance Christian Hospital, Wonju, South Korea; ^3^ Division of Metabolism, Endocrine, and Diabetes, University of Michigan, Ann Arbor, MI, United States

**Keywords:** primary aldosteronism, aldosterone, angiotensin, adrenocorticotropic hormone (ACTH), adrenal, adrenal cortex

## Abstract

**Background:**

Somatic gene mutations that facilitate inappropriate intracellular calcium entrance have been identified in most aldosterone-producing adenomas (APAs). Studies suggest that angiotensin II and adrenocorticotropic hormone (ACTH) augment aldosterone production from APAs. Little is known, however, regarding possible variations in response to hormonal stimuli between APAs with different aldosterone-driver mutations.

**Objective:**

To analyze the transcript expression of type 1 angiotensin II receptors (*AGTR1*), ACTH receptors (*MC2R*), and melanocortin 2 receptor accessory protein (*MRAP*) in APAs with known aldosterone-driver somatic mutations.

**Methods:**

RNA was isolated from APAs with mutations in: *KCNJ5* (n = 14), *ATP1A1* (n = 14), *CACNA1D* (n = 14), and *ATP2B3* (n = 5), and from normal adjacent adrenal tissue (n = 45). Transcript expression of *MC2R*, *MRAP*, *AGTR1*, aldosterone synthase (*CYP11B2*), 17α-hydroxylase/17,20-lyase (*CYP17A1*), and 11β-hydroxylase (*CYP11B1*) were quantified using quantitative RT-PCR and normalized to β-actin.

**Results:**

Compared to adjacent normal adrenal tissue, APAs had higher transcript levels of *CYP11B2* (2,216.4 [1,112.0, 2,813.5]-fold, *p *< 0.001), *MC2R* (2.88 [2.00, 4.52]-fold, *p* < 0.001), and *AGTR1* (1.80 [1.02, 2.80]-fold, *p* < 0.001]), and lower transcript levels of *MRAP*, *CYP17A1*, and *CYP11B1* (0.28–0.36, *p* < 0.001 for all). *MC2R* and *CYP11B2* transcripts were lower in APAs with *KCNJ5 vs.* other mutations (*p* < 0.01 for both). *MC2R* expression correlated positively with that of *AGTR1* in APAs harboring *KCNJ5* and *CACNA1D* mutations, and with *MRAP* expression in APAs harboring *ATP*ase mutations.

**Conclusions:**

While *MC2R* and *AGTR1* are expressed in all APAs, differences were observed based on the underlying aldosterone-driver somatic mutations. In tandem, our findings suggest that APAs with *ATPase*-mutations are more responsive to ACTH than *KCNJ5*-mutated APAs.

## Introduction

Primary aldosteronism (PA) is characterized by inappropriate, renin-independent aldosterone production. PA is the most common curable form of secondary hypertension, accounting for up to 20% of resistant hypertension cases (1). Growing evidence suggests that PA increases the risk of cardiovascular and renal complications as compared to essential hypertension, independently of blood pressure control (2–4). Inappropriate mineralocorticoid receptor activation might promote the release of pro-inflammatory cytokines (5), oxidative stress (6), and, consequently, target organ damage (2, 4). Sporadic PA is broadly classified as bilateral adrenal hyperaldosteronism (BHA) or unilateral PA, which is often caused by an aldosterone-producing adenoma (APA). APAs account for 30–50% of PA cases and they can be cured by adrenalectomy, while BHA requires life-long targeted medical therapy (7). PA subtyping is typically established based on adrenal venous sampling (AVS) (7). In many centers, AVS is performed after administration of cosyntropin, a synthetic adrenocorticotropic hormone (ACTH), which enhances the confidence of successful adrenal vein catheterization and circumvents intrinsic ACTH fluctuations that might occur due to the stress of the procedure. Reports regarding the impact of ACTH on APAs, however, have been inconsistent (8–12).

Studies conducted over the past decade have identified a series of aldosterone–driver gene mutations in familial and sporadic forms of PA. Affected genes include: *KCNJ5* (13), *ATP1A1* (14, 15), *ATP2B3* (15), *CACNA1D* (16)*, CACNA1H* (17), *CTNNB1* (18), and *CLCN2* (19, 20). Next-generation sequencing (NGS) of aldosterone-producing areas precisely mapped using immunohistochemistry (IHC) for aldosterone synthase (CYP11B2) has revealed aldosterone-driver somatic mutations in over 90% of APAs (21–23). A shared molecular feature of the somatic mutations found in APAs is that they facilitate intracellular calcium entrance, which then stimulates aldosterone production by augmenting CYP11B2 expression (23). Nonetheless, APAs harboring different aldosterone-driver somatic mutations have distinct histopathological features (24), steroidogenic potential (25), and responses to ACTH stimulation (26).

In addition to ion channel or pump mutations, some studies suggest that the aberrant expression of receptors in APAs, such as G-protein coupled receptors (GPCRs), might contribute to their dysregulated aldosterone production (27–29). Under physiological conditions, angiotensin II, serum potassium, and, to a lesser extent, ACTH control aldosterone synthesis from the adrenal zona glomerulosa (ZG) (30, 31). Variability in type 1 angiotensin II receptor (*AGTR1*) and melanocortin type 2 receptor (*MC2R*, also known as ACTH receptor) expression, which is abundant in both APAs and normal adrenals (29), might modulate aldosterone production (30, 31). Although cellular models of aldosterone-driver mutations showed that responses to angiotensin II are increased (32, 33), data on possible variations in response to hormonal stimuli between APAs with different somatic mutations are scarce. Herein, we investigated the transcript expression of *AGTR1*, *MC2R*, and melanocortin-2-receptor accessory protein (*MRAP*) in APAs with known aldosterone-driver somatic mutations and in adjacent normal adrenal tissue. In addition, we assessed the relationship between aldosterone-regulators and *CYP11B2* expression in APAs with different somatic mutations.

## Materials and Methods

### Tissue Samples

The current study included adrenals from 47 patients with APA who underwent adrenalectomy at the University of Michigan between 2004 and 2018. Patients were selected based on availability of formalin-fixed paraffin-embedded (FFPE) adrenal tumor blocks. The clinical diagnosis of PA was made according to the institutional consensus available at the time or the Endocrine Society Clinical Practice guidelines (7). All adrenal specimens were pathologically diagnosed as adrenocortical adenomas. For comparison, we used adjacent normal adrenal tissue obtained from the same patients. Because the availability of adrenal tissue adjacent to the APA was limited, cortical and medullary tissue were not dissected separately. Sections from FFPE adrenal tumor blocks were used for IHC for CYP11B2 and 17α-hydroxylase/17,20-lyase (CYP17A1) and for genetic analysis, as previously described (21). This study was approved by Institutional Review Boards at the University of Michigan (HUM00106809, HUM00024461, HUM00083056). Written informed consent was obtained from all patients who underwent adrenalectomy after February, 2011. A waiver of consent was granted for the use of archival specimens (HUM00083056).

### DNA/RNA Isolation

Genomic DNA (gDNA) and RNA were obtained from APAs with mutations in: *KCNJ5* (n = 14), *ATP1A1* (n = 14), *CACNA1D* (n = 14), and *ATP2B3* (n = 5), and from adjacent normal adrenal tissues (n = 45). Adrenocortical adenomas that displayed CYP11B2-expressing cells were considered APAs. After identification of CYP11B2-positive areas by IHC, four to nine unstained consecutive 5 µm FFPE slides were used to separately dissect corresponding CYP11B2-positive areas. Dissection of FFPE sections was performed using disposable scalpels under an Olympus SZ-40 microscope. The AllPrep DNA/RNA FFPE kit (QIAGEN, Hilden, Germany) was used to isolate gDNA and RNA, as previously described (34).

### Next-Generation Sequencing

For mutation analysis, multiplexed PCR–based NGS was conducted using Ion Torrent Ampliseq sequencing (Thermo Fisher Scientific), as previously described (21, 34). The panel for library preparation included amplicons targeting the full coding regions of known aldosterone-driving genes, including the most commonly affected: *KCNJ5*, *ATP1A1*, *CACNA1D*, and *ATP2B3*. APAs with other aldosterone-driver mutations were not included in this analysis, due to their low prevalence.

### Quantitative Real-Time RT-PCR (qPCR)

Total RNA was reverse transcribed using the High-Capacity cDNA Reverse Transcription Kit (Applied Biosystems). qPCR was performed using the ABI StepOnePlus Real-Time PCR systems (Applied Biosystems). *CYP11B2*, *CYP17A1*, and *CYP11B1* primer/probe mixtures were prepared as previously described (27, 35). For Human *MRAP* qPCR, the primer (qHsaCID0022591, Bio-Rad) was mixed with SYBR Green PCR master mix (Applied Biosystems). Primer/probe mixtures for the amplification of *AGTR1* (Hs00258938_m1), *MC2R* (Hs00300820_s1), and β-actin (*ACTB*; Hs01060665_g1) were purchased from Applied Biosystems. In this study, *ACTB* transcript was used as a reference gene for normalization between samples. Relative quantification was determined using the comparative threshold cycle method (36). The average ΔCT value of all adjacent normal tissues was used as reference when comparing gene expression between APAs with various underlying mutations.

### Statistical Analysis

Statistical analyses were conducted using SAS 9.4 (SAS Institute, Cary, NC, USA), and GraphPad Prism 8 was used to generate figures. The Kruskal-Wallis test, followed by the Dwass-Steel-Critchlow-Fligner test were employed to compare continuous variables across multiple groups. Distribution of categorical variables across groups was assessed by the Chi-square or Fisher’s exact test. Wilcoxon signed-rank test was used for paired comparison of transcript levels between APAs and the corresponding adjacent normal adrenal tissues. Correlations between gene expressions were examined with the Spearman correlation test. Two-sided *p* values below 0.05 were considered statistically significant.

## Results

Demographic and clinical characteristics of study participants are presented in **Table 1**. Most patients were Caucasian, with ages between 20 and 79 years (median age 52) and 62% were men. Patients with APAs harboring *KCNJ5* mutations were younger, leaner, and mostly women (**Table 1**).

**Table 1:** Baseline characteristics of patients with APA participating in this study.

	Total (n = 47)	*KCNJ5 *(n = 14)	*ATP1A1 *(n = 14)	*CACNA1D *(n = 14)	*ATP2B3 *(n = 5)	*p* value
Age (years)	52.0 (20, 79)	42.0 (20, 56)	55.5 (41, 79)	53.0 (32, 78)	59.0 (53, 75)	0.002
Sex (n men, %)	29 (61.7%)	1 (7.1%)	12 (85.7%)	11 (78.6%)	5 (100%)	<0.001
Race (n)	C (38), AA (4), A (1), U (4)	C (10), AA (1), A (1), U (2)	C (13), U (1)	C (11), AA (2), U (1)	C (4), AA (1)	0.496
BMI (kg/m^2^) [n = 33]	30.6 [26.2, 35.7]	25.2 [23.2, 33.4]	34.7 [31.9, 40.6]	30.6 [26.8, 33.9]	29.1 [26.1, 30.6]	0.024
SBP (mmHg) [n = 44]	145.5 [130.3, 167.5]	141.0 [128.0, 175.0]	158.5 [130.5, 182.0]	145.0 [134.3, 159.8]	149.0 [135.5, 165.5]	0.779
DBP (mmHg) [n = 44]	86.0 [74.0, 91.8]	76.0 [70.0, 92.5]	90.0 [83.0, 96.3]	85.5 [74.5, 98.0]	78.0 [73.0, 84.5]	0.270
Serum Cr (mg/dl) [n = 30]	0.90 [0.79, 1.10]	0.78 [0.69, 0.90]	0.94 [0.81, 1.09]	1.03 [0.83, 1.23]	1.50 [1.20, 3.43]	0.003
Serum potassium (mmol/L) [n = 43]	3.4 [2.9, 3.8]	3.3 [2.9, 3.9]	3.4 [2.9, 3.7]	3.6 [3.4, 3.8]	3.2 [3.0, 3.9]	0.462
PAC (ng/dl) [n = 44]	29.1 [21.7, 60.2]	26.2 [19.6, 36.1]	29.7 [23.3, 98.4]	27.4 [21.7, 48.1]	80.0 [27.1, 230.0]	0.296
PRA (ng/ml/hr) [n = 31]	0.20 [0.10, 0.60]	0.10 [0.07, 0.60]	0.10 [0.10, 0.40]	0.30 [0.15, 0.75]	0.30 [0.10, 0.73]	0.399

Continuous variables are expressed as median [interquartile range], except for age, which is expressed as median (range).

APA, aldosterone-producing adenoma; C, Caucasian; AA, African American; A, Asian; U, unknown; BMI, body mass index; SBP, systolic blood pressure; DBP, diastolic blood pressure; Cr, creatinine; PAC, plasma aldosterone concentration; PRA, plasma renin activity.

### AGTR1, MC2R, MRAP, CYP11B2, CYP17A1, and CYP11B1 Gene Expressions in Aldosterone-Producing Adenomas

Overall, APAs displayed higher transcript levels of *MC2R* (2.88 [2.00, 4.52]-fold, *p *< 0.001), *AGTR1* (1.80 [1.02, 2.80]-fold, *p* < 0.001), and *CYP11B2* (2216.4 [1112.0, 2813.5]-fold, *p* < 0.001) compared to the corresponding adjacent normal adrenal tissue, and these differences remained robust in APAs with *CACNA1D* and *ATP1A1* mutations (**Table 2**). *AGTR1* and *MC2R* transcript levels were only minimally, but not significantly higher in *KCNJ5*-mutated APAs as compared to the paired adjacent normal adrenal tissue. Conversely, APAs had lower transcript levels of *MRAP*, *CYP17A1*, and *CYP11B1* (0.28–0.36-fold, *p* < 0.001, **Table 2**) than the corresponding normal adjacent adrenal tissue and these differences were observed in all mutation subgroups.

**Table 2:** Paired comparisons of transcript levels of *AGTR1*, *MC2R*, *MRAP*, and steroidogenic enzymes between APAs and adjacent normal adrenal tissue.

	*AGTR1*	*MC2R*	*CYP11B2*	*MRAP*	*CYP17A1*	*CYP11B1*
***All APAs***						
APAs	1.80 [1.02, 2.80]	2.88 [2.00, 4.52]	2,216.40 [1,111.98, 2,813.45]	0.36 [0.18, 0.59]	0.30 [0.15, 0.43]	0.28 [0.19, 0.56]
Adjacent adrenal tissue	0.99 [0.64, 1.49]	0.99 [0.65, 1.43]	1.07 [0.35, 2.70]	0.97 [0.64, 1.65]	0.99 [0.78, 1.31]	1.04 [0.78, 1.27]
***p* value**	<0.001	<0.001	<0.001	<0.001	<0.001	<0.001
***KCNJ5-mutated APAs***						
APAs	1.37 [1.06, 2.11]	1.90 [1.13, 2.52]	911.30 [502.92, 1,212.01]	0.41 [0.23, 0.66]	0.32 [0.28, 0.53]	0.53 [0.20, 0.81]
Adjacent adrenal tissue	0.99 [0.58, 1.35]	0.98 [0.53, 1.80]	0.38 [0.15, 2.31]	1.15 [0.83, 1.85]	0.92 [0.80, 1.17]	1.06 [0.75, 1.30]
***p* value**	0.101	0.064	0.001	0.004	0.002	0.002
***CACNA1D-mutated APAs***						
APAs	2.25 [1.52, 2.89]	3.48 [2.57, 4.36]	2,559.10 [1,506.43, 3,273.80]	0.32 [0.12, 0.58]	0.28 [0.19, 0.45]	0.20 [0.15, 0.36]
Adjacent adrenal tissue	1.14 [0.79, 1.61]	1.08 [0.67, 1.51]	1.09 [0.49, 2.20]	1.29 [0.89, 2.36]	1.34 [0.90, 1.49]	1.04 [0.77, 1.20]
***p* value**	0.013	0.001	0.001	0.001	0.001	0.001
***ATP1A1-mutated APAs***						
APAs	1.57 [0.98, 3.01]	5.13 [2.35, 7.55]	2,329.07 [1,519.96, 4,213.90]	0.43 [0.22, 0.58]	0.18 [0.10, 0.40]	0.31 [0.25, 0.48]
Adjacent adrenal tissue	1.16 [0.65, 1.56]	1.16 [0.73, 1.36]	1.59 [0.87, 7.77]	0.66 [0.60, 1.17]	0.85 [0.67, 1.24]	0.96 [0.76, 1.66]
***p* value**	0.013	0.001	0.001	0.002	0.001	0.001
***ATP2B3-mutated APAs***						
APAs	2.91 [1.02, 6.97]	4.18 [2.58, 6.34]	2,736.94 [1,755.25, 4,163.27]	0.36 [0.14, 0.63]	0.20 [0.03, 0.49]	0.19 [0.16, 0.55]
Adjacent adrenal tissue	0.69 [0.57, 0.98]	0.73 [0.48, 1.14]	0.51 [0.25, 1.94]	0.65 [0.52, 0.78]	0.88 [0.72, 1.04]	1.07 [0.73, 1.09]
***p* value**	0.144	0.068	0.068	0.068	0.068	0.068

qPCR data are shown as fold changes normalized to β-actin (ACTB). Continuous variables are expressed as median [interquartile range].

APA, aldosterone-producing adenoma; AGTR1, type 1 angiotensin II receptor; MC2R, melanocortin type 2 receptors (ACTH receptors); CYP11B2, aldosterone synthase; MRAP, melanocortin 2 receptor accessory protein; CYP17A1, 17α-hydroxylase; CYP11B1, 11β-hydroxylase.

**Figure 1:**
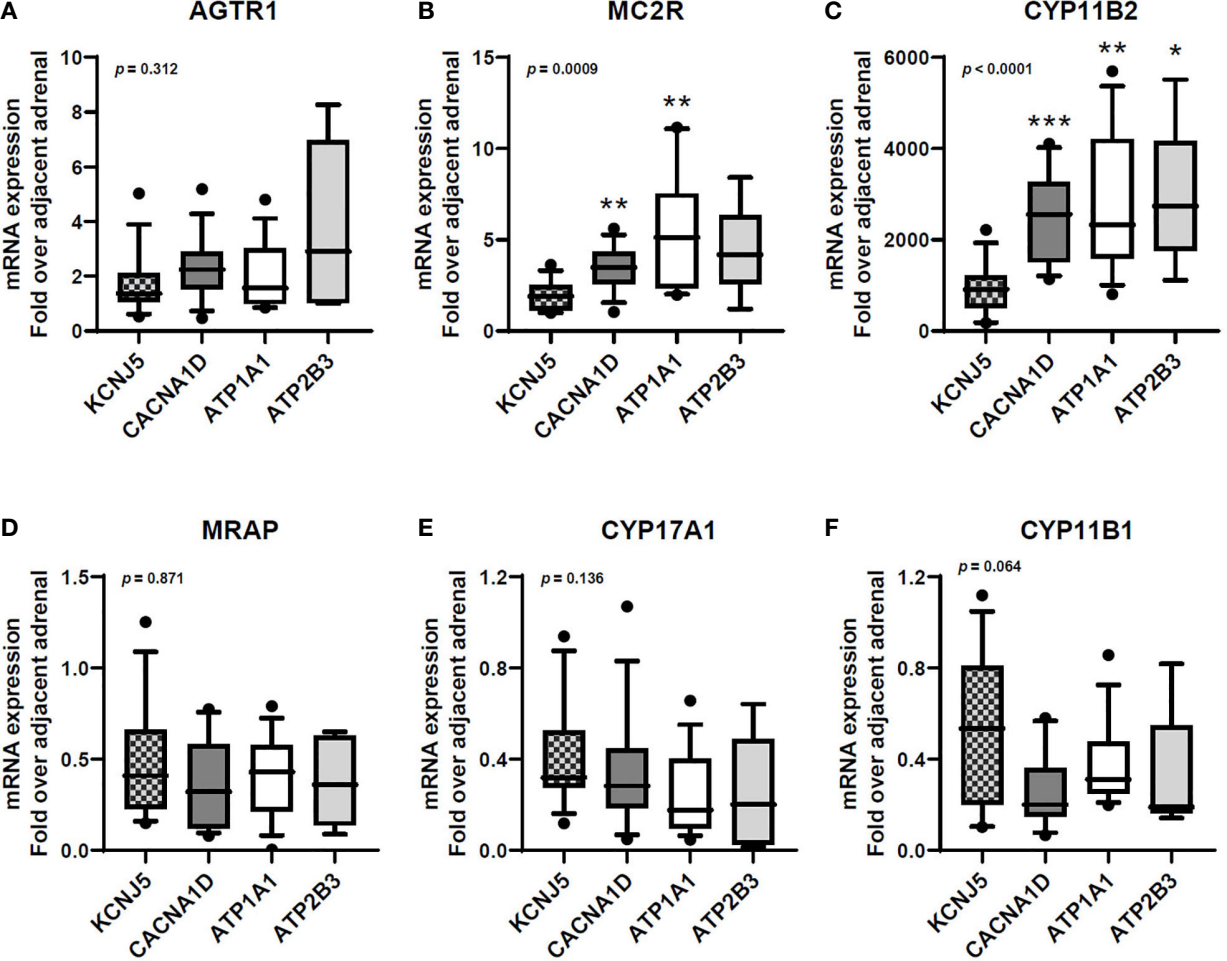
Transcript expression of *AGTR1* **(A)**, *MC2R* **(B)**, *CYP11B2* **(C)**, *MRAP* **(D)**, *CYP17A1* **(E)**, and *CYP11B1* **(F)** in aldosterone-producing adenomas with different aldosterone-driver somatic mutations. qPCR data are shown as the fold changes normalized to β-actin (ACTB). *AGTR1*, type 1 angiotensin II receptor; *MC2R*, melanocortin type 2 receptors (ACTH receptors); *CYP11B2*, aldosterone synthase; *MRAP*, melanocortin 2 receptor accessory protein; *CYP17A1*, 17α-hydroxylase; *CYP11B1*, 11β-hydroxylase. Comparisons between groups were done using the Kruskal-Wallis test, followed by the Dwass-Steel-Critchlow-Fligner test. *p < 0.05, **p < 0.01, ***p < 0.001, compared with *KCNJ5*-mutated APAs. The boxes contain the 25^th^ and 75^th^ percentiles, the whiskers mark the 10^th^ and 90^th^ percentiles, and the horizontal line within the box indicates the median, and the ⚫ represent outliers.

APAs harboring *KCNJ5* mutations displayed lower *MC2R* and *CYP11B2* mRNA expressions compared to other APAs (**Figures 1B, C**), while *AGTR1* and *MRAP* transcript levels were relatively similar between mutation groups (**Figures 1A, D**).

### Correlations Between Aldosterone Regulators and Steroidogenic Enzymes in Aldosterone-Producing Adenomas

**Figure 2:**
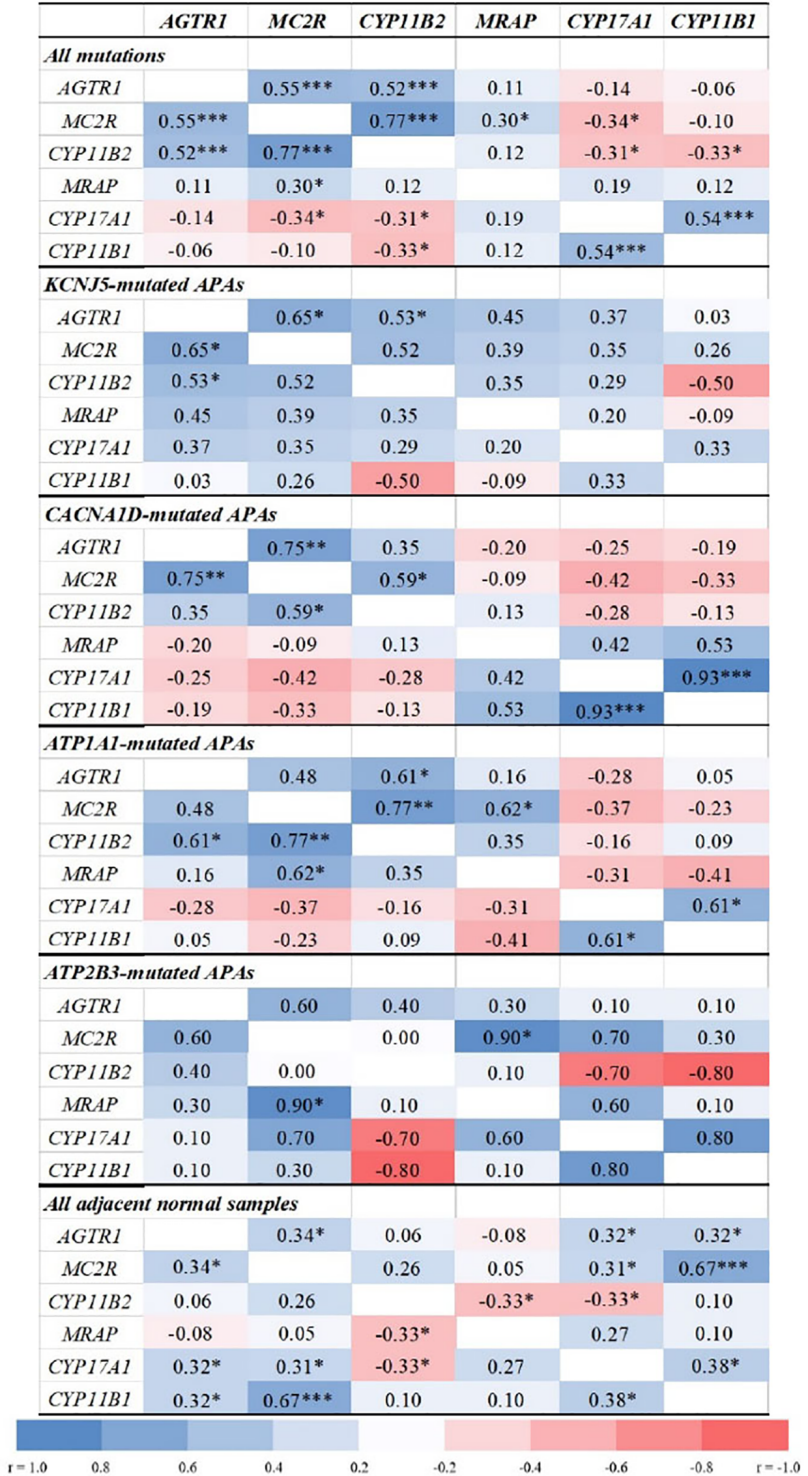
Correlations between transcript levels of *AGTR1*, *MC2R*, *MRAP*, and steroidogenic enzymes in aldosterone-producing adenomas and adjacent normal adrenal tissue. *AGTR1*, type 1 angiotensin II receptor; *MC2R*, melanocortin type 2 receptors (ACTH receptors); *CYP11B2*, aldosterone synthase; *MRAP*, melanocortin 2 receptor accessory protein; *CYP17A1*, 17α-hydroxylase; *CYP11B1*, 11β-hydroxylase. Correlation analyses were done using the Spearman correlation test. *p < 0.05, **p < 0.01, ***p < 0.001.

Overall, APA *CYP11B2* expression correlated positively with *MC2R* (r = 0.77, *p* < 0.0001) and *AGTR1* (r = 0.52, *p* = 0.0002, **Figure 2**), and inversely with *CYP17A1* and *CYP11B1* (r = −0.3, *p *< 0.05 for both). The strongest correlations between *CYP11B2* and both *MC2R* and *AGTR1* were observed in *ATP1A1-*mutated APAs (r = 0.77, *p* = 0.001 and r = 0.61, *p* = 0.021, respectively).

APAs with *CACNA1D* and *KCNJ5* mutations displayed tight positive correlations between *MC2R* and *AGTR1* transcripts (r = 0.75, *p* = 0.002 and r = 0.65, *p* = 0.012, respectively), while no significant correlations were found in APAs with *ATPase* mutations. Conversely, *MC2R* and *MRAP* expressions correlated positively only in *ATP1A1*- and *ATP2B3*-mutated APAs (r = 0.62, *p* = 0.018 and r = 0.90, *p* = 0.037, respectively).

## Discussion

In this study, we delineate differential gene expression of the primary aldosterone regulatory receptors in APAs with different underlying mutations. We found that APAs displayed higher mRNA expression of both *MC2R* and *AGTR1* than adjacent normal adrenal tissue. In addition, we show that the expression patterns of *MC2R* and *AGTR1*, and their associations with *CYP11B2* transcripts differ between APAs with various underlying aldosterone-driver somatic mutations.

Under physiological conditions, angiotensin II induces Gi-mediated cell membrane depolarization and increases intracellular calcium signaling, thereby stimulating acute steroid production as a result of increased steroidogenic acute regulatory protein (StAR) protein expression (31). Furthermore, this elevation in intracellular calcium activates a cascade of signaling events that lead to increased *CYP11B2* transcription and aldosterone secretion from ZG cells (30, 37). Although PA is theoretically renin-independent, aldosterone excess may also result from aberrant receptor expression within APAs and/or hypersensitivity to physiological stimuli. A variety of autocrine and paracrine regulatory factors (38) can activate ectopic or aberrant receptors, which may govern aldosterone secretion independently from the suppressed renin-angiotensin system (29, 39). Indeed, mRNA expressions of *AGTRI* and *MC2R* were previously reported to be higher in APA tissues compared to healthy adult adrenals (27, 29, 40). The effects of posture, angiotensin II infusion, and angiotensin converting enzyme inhibitors have been shown to differ in APA when compared to BHA, although results have been variable (7, 41–43). In our study, *AGTR1* transcript levels tended to be higher in APAs as compared to adjacent normal adrenal tissue. Tunny and colleagues found that angiotensin II-unresponsive APAs were more common in women, while those responsive to angiotensin II were more prevalent in men (41). Indeed, we herein found that *KCNJ5*-mutated APAs, which are most prevalent in women of all races (44–46), expressed *AGTR1* transcript levels comparable to those found in the corresponding normal adrenal tissue.

In contrast with angiotensin II and potassium, ACTH stimulates aldosterone secretion acutely but transiently (31, 47). Aldosterone production follows a circadian rhythm that parallels that of ACTH both in normal individuals, as well as in patients with PA (48, 49). In patients with aldosterone-secreting tumors, plasma aldosterone concentration starts to fall around mid-morning, as ACTH levels decrease, in spite of upright posture (39). The relative impact of ACTH on aldosterone production from APA *vs.* BHA and normal ZG cells remains incompletely understood. Small studies suggest that APAs might be more sensitive to ACTH stimulation and suppression than BHA and normal adrenals (49). Asian studies (50–52) indicated that the response of aldosterone to cosyntropin stimulation, with or without *a priori* overnight suppression with 1mg dexamethasone, is higher in patients with APA than in those with BHA. Nevertheless, AVS data have shown that aldosterone lateralization might be apparent only prior to or exclusively after cosyntropin stimulation (8, 9, 53). Washout of a baseline aldosterone gradient between the two adrenal glands following cosyntropin stimulation indicates a relatively higher response from either normal ZG cells or from asymmetrical BHA. Conversely, amplification of a baseline aldosterone lateralization points towards a highly ACTH-sensitive APA.

The impact of ACTH on aldosterone secretion is dependent on the expression of *MC2R* in CYP11B2-positive cells (31). As ACTH is the primary regulator of cortisol synthesis, *MC2R* is abundantly expressed in the zona fasciculata (ZF) cells (54). Previous studies have shown that APAs have higher *MC2R* transcript levels than normal adrenal tissue, non-functional adrenal adenomas, or carcinomas (27, 29, 40, 55–57), although the levels reported have been somewhat variable. Our study is the first to quantify the expression of *MC2R* and *AGTR1* transcript levels in APAs confirmed by CYP11B2 IHC. Non-functional cortical adenomas can be present in patients with PA, and these tumors display lower *MC2R* expression than APAs or normal cortical tissue (40, 55); this might explain previously reported variability of *MC2R* expression in presumed APAs that were not functionally confirmed by examining CYP11B2 expression. Another cause of variability relates to the APA genotype. While all APAs had higher transcript levels of *MC2R* compared to adjacent normal adrenal tissue*, KCNJ5-*mutated APAs displayed lower *MC2R* transcripts than other APAs. Considering that BHA are often caused by multiple APCCs that harbor *CACNA1D* mutations (58), it is not surprising that East Asians studies that assessed the aldosterone response to ACTH stimulation or suppression in patients with APA *vs.* BHA found considerable overlap. As confirmed by several cohorts, *KCNJ5* mutations account for the vast majority of APAs in East Asian populations (45, 59). In line with these findings, we have previously reported that aldosterone lateralization during AVS often dampens following cosyntropin stimulation in patients with APAs harboring *KCNJ5* mutations, while the opposite happens in patients with *ATPase* mutations (26).

ACTH binds to its MC2R, and induces the activation of adenylate cyclase and the generation of intracellular cAMP (54, 60). Subsequently, the increased cAMP activates protein kinase A, which augments CREB phosphorylation and *CYP11B2* transcription (30, 31). MRAP, a small transmembrane protein, is an essential factor in regulating trafficking and functional expression of the MC2R in the adrenal gland (61, 62). Both *MC2R* and *MRAP* are known to be highly expressed in the undifferentiated zone as well as the ZF cells (63). Furthermore, the acute steroidogenic responses to ACTH stimulation depend on adequate amounts of *MC2R* and *MRAP* on the plasma membrane surface (61). In this study, *MC2R* transcripts correlated positively with *MRAP* expression only in *ATPase-*mutated APAs. These findings further support the high responsivity of *ATPase*-mutated APAs to cosyntropin observed during AVS (26), in contrast with *KCNJ5* or *CACNA1D*-mutated APAs. Conversely, *MC2R* transcript levels correlated positively with those of *AGTR1* in APAs harboring *KCNJ5* or *CACNA1D* mutations, but not in those with *ATPase* mutations. Together these results highlight molecular differences between APAs, which go beyond those illustrated by recent histopathological studies (23, 24). Additional downstream molecular mechanisms might be impacted differently by various aldosterone-driver mutations and deserve further investigation. For example, *in vitro* studies suggest that angiotensin II upregulates *NR4A1*, *NR4A2*, and *NR4A3* gene expression (64, 65), and that *NR4A2* and *NR4A3* are upregulated in cell models overexpressing *KCNJ5* mutations (66, 67). Other transcriptome and methylome variations have been shown between APA with and without KCNJ5 mutations (68). In addition, differences in the expression of inhibitory regulators, such as dopamine receptors (69, 70) across APAs with various aldosterone-driver mutations deserve further investigation.

In summary, we found that ACTH and angiotensin II receptors are expressed in functionally confirmed APAs harboring the four most common aldosterone-driver somatic mutations. Additionally, we show that these key aldosterone regulatory receptors display several differences in expression across APAs with distinct underlying mutations. Specifically, *KCNJ5*-mutated APAs express lower mRNA transcript levels of both *MC2R* and *CYP11B2* as compared to other APAs, and they display no association between *MC2R* and *MRAP* expression, possibly explaining their relatively modest response to cosyntropin stimulation observed during AVS. Conversely, *ATP1A1*-mutated APAs showed robust positive correlation of *MC2R* with both *MRAP* and *CYP11B2* expression, supporting their ACTH-sensitivity. The relatively small number of tissue samples and individual variability from APAs with distinct somatic mutation are limitation of our study. Another important limitation is the lack of protein translation assessment, and thus conclusions regarding protein function remain limited. Such studies will be critical once highly selective human MC2R antibodies become available. Nevertheless, this initial study provides insight into the possible actions of ACTH and angiotensin II in APA with various aldosterone-driver mutations.

## Data Availability Statement

The original contributions presented in the study are included in the article/supplementary material. Further inquiries can be directed to the corresponding author.

## Ethics Statement

This research was reviewed and approved by the Institutional Review Boards at the University of Michigan (HUM00106809, HUM00024461, HUM00083056). Written informed consent was obtained from all patients who underwent adrenalectomy prior to February, 2011. A waiver of consent was granted for the use of archival specimens (HUM00083056).

## Author Contributions

JSL, WER, and AFT conceived and designed the study. JSL and SP performed the experiment. JSL and AFT analyzed the data. JSL, JR, WR, and ADT interpreted the data. JL and AFT drafted and revised the manuscript. All authors contributed to the article and approved the submitted version.

## Funding

AFT was supported by grants 1K08DK109116 from the NIDDK and DDCF_2019087 from the Doris Duke Charitable Foundation. WER was supported by grant R01DK106618 from the NIDDK.

## Conflict of Interest

The authors declare that the research was conducted in the absence of any commercial or financial relationships that could be construed as a potential conflict of interest.

The reviewer SM declared a past co-authorship with one of the authors WR to the handling editor.
